# Recent Advances in Deep Learning and Medical Imaging for Head and Neck Cancer Treatment: MRI, CT, and PET Scans

**DOI:** 10.3390/cancers15133267

**Published:** 2023-06-21

**Authors:** Mathew Illimoottil, Daniel Ginat

**Affiliations:** 1 School of Medicine, University of Missouri-Kansas City, Kansas City, MO 64018, USA; 2 Department of Radiology, The University of Chicago, Chicago, IL 60637, USA

**Keywords:** magnetic resonance imaging (MRI), computed tomography (CT), positron emission tomography (PET), deep learning, radiomics, radiogenomics, convolutional neural networks (CNNs), artificial intelligence (AI), generative adversarial networks (GANs), head and neck, cancer, treatment

## Abstract

**Simple Summary:**

Deep learning techniques have significant potential in head and neck cancer imaging, particularly in tumor detection, segmentation, and outcome prediction using magnetic resonance imaging (MRI), computed tomography (CT), and positron emission tomography (PET) scans. Advanced deep learning methods, such as convolutional autoencoders, generative adversarial networks (GANs), and transformer models, have further enhanced imaging capabilities. Comparing deep learning and traditional techniques and the advantages and limits of each reveals their complementary roles in cancer management. Integrating radiogenomics with deep learning models promises further advancements in personalized care. However, challenges such as standardization, data quality, model overfitting, and computational requirements persist. Addressing these issues, integrating multimodal and temporal information, enhancing explainability, and conducting clinical validation are crucial for implementing deep learning models in head and neck cancer diagnosis and treatment. Overcoming these obstacles will pave the way for improved patient outcomes and personalized treatment strategies in head and neck cancer management.

**Abstract:**

Deep learning techniques have been developed for analyzing head and neck cancer imaging. This review covers deep learning applications in cancer imaging, emphasizing tumor detection, segmentation, classification, and response prediction. In particular, advanced deep learning techniques, such as convolutional autoencoders, generative adversarial networks (GANs), and transformer models, as well as the limitations of traditional imaging and the complementary roles of deep learning and traditional techniques in cancer management are discussed. Integration of radiomics, radiogenomics, and deep learning enables predictive models that aid in clinical decision-making. Challenges include standardization, algorithm interpretability, and clinical validation. Key gaps and controversies involve model generalizability across different imaging modalities and tumor types and the role of human expertise in the AI era. This review seeks to encourage advancements in deep learning applications for head and neck cancer management, ultimately enhancing patient care and outcomes.

## 1. Introduction

Head and neck cancers represent a heterogeneous group of malignancies that arise from various structures in the head and neck region, including the oral cavity, pharynx, larynx, nasal cavity, paranasal sinuses, and salivary glands [1]. These cancers are often associated with significant morbidity and mortality, and their management requires a multidisciplinary approach involving surgery, radiation therapy, and chemotherapy [2]. Accurate diagnosis, staging, and monitoring of head and neck cancers are crucial for determining the optimal treatment strategy, predicting patient outcomes, and assessing treatment response.

Medical imaging techniques, such as magnetic resonance imaging (MRI), computed tomography (CT), and positron emission tomography (PET) scans, have been indispensable in the assessment of head and neck cancers [3]. However, the interpretation of these images can be challenging due to the complex anatomy, diverse tumor biology, and overlapping features of malignant and benign lesions [4]. Moreover, traditional imaging techniques often rely on human expertise, which may be subjective and prone to variability.

Deep learning algorithms, particularly convolutional neural networks (CNNs), have shown potential in improving the accuracy, efficiency, and consistency of medical image analysis [5]. These advanced computational techniques can automatically learn hierarchical feature representations from raw imaging data, enabling the extraction of clinically relevant information and potentially surpassing human-level performance in certain tasks.

In this review, we delve into the limitations of traditional imaging techniques in head and neck cancer imaging and discuss advanced deep learning techniques, such as convolutional autoencoders for data compression, image denoising, and feature extraction; generative adversarial networks (GANs) for super-resolution; and transformer models such as the Vision Transformer (ViT). We also compare deep learning and traditional imaging techniques in head and neck cancer imaging, emphasizing their complementary roles and the potential benefits of integrating these methods.

In recent years, deep learning applications have expanded to include tumor detection, segmentation, classification, and response prediction, as well as the integration of multi-modal imaging data and the development of radiomics-based predictive models [6]. We explore the potential of deep learning to advance precision medicine in head and neck cancer management by enabling personalized treatment planning and outcome predictions. Additionally, we examine the emerging field of radiogenomics, which utilizes information about the relationship between imaging features and genomic information to create predictive models that can better guide medical treatment. We identify gaps in current research and propose future directions that may further enhance the impact of deep learning on the diagnosis and treatment of head and neck cancers.

This review aims to inform researchers and clinicians about the progress, potential, and obstacles in the application of deep learning techniques in head and neck cancer imaging, ultimately guiding future research toward improving patient outcomes and personalized treatment strategies. By incorporating the latest advancements and a comprehensive comparison of traditional and deep learning techniques, we provide a concise but informative understanding of the current landscape and future prospects in head and neck cancer imaging.

## 2. Limitations of Traditional Imaging Techniques in Head and Neck Cancer Imaging

Traditional imaging techniques, such as magnetic resonance imaging (MRI), computed tomography (CT), and positron emission tomography (PET) scans, play crucial role sin the diagnosis, staging, and monitoring of head and neck cancers [7]. However, these methods often rely on human expertise for image interpretation, which may be subjective and prone to variability [8]. In this section, we discuss the limitations of traditional imaging techniques in head and neck cancer imaging, focusing on the challenges associated with human expertise and the potential consequences for clinical decision-making.

### 2.1. Subjectivity and Interobserver Variability

Radiologists may assess medical images based on qualitative criteria, such as lesion shape, size, or intensity, which can lead to inconsistencies in diagnosis and staging among different observers [9]. For example, in the case of head and neck cancers, the complex anatomy and overlapping features of malignant and benign lesions can make accurate interpretation of imaging data challenging, even for experienced radiologists [10]. Additionally, manual delineation of tumor boundaries in imaging data can be time-consuming, and interobserver variability can affect the accuracy and reliability of the results [11].

### 2.2. Factors Affecting Human Expertise

Human interpretation of medical images may be influenced by factors such as fatigue, experience, and cognitive biases, which can contribute to discrepancies in decision-making [12]. For instance, studies have shown that radiologists’ diagnostic performance can be affected by factors such as workload, time constraints, and confirmation bias, leading to potential errors in cancer detection and staging [13,14]. Furthermore, the lack of standardized guidelines and protocols for image interpretation in head and neck cancer imaging can exacerbate these issues, resulting in suboptimal clinical decision-making and patient management.

### 2.3. Need for Improved Imaging Techniques

Given the limitations of traditional imaging techniques and the potential impact on patient outcomes, there is a growing need for more accurate, efficient, and consistent methods for head and neck cancer imaging. Deep learning algorithms, particularly convolutional neural networks (CNNs), offer a promising solution to these challenges since they can automatically learn hierarchical feature representations from raw imaging data and potentially surpass human-level performance in certain tasks [15,16]. In the following sections, we discuss the recent advances in deep learning applications for head and neck cancer treatment, focusing on the use of MRI, CT, and PET scans.

## 3. Deep Learning in Medical Imaging

### 3.1. Deep Learning: A Brief Overview

**Figure 1:**
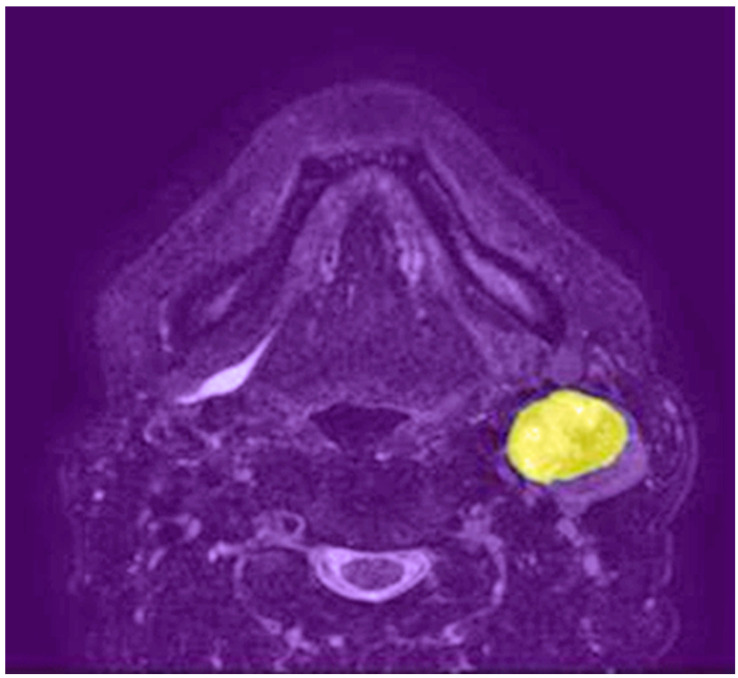
Axial T2-weighted MRI shows a left neck lymph node metastasis segmented by a neural network algorithm (highlighted in yellow).

**Figure 2:**
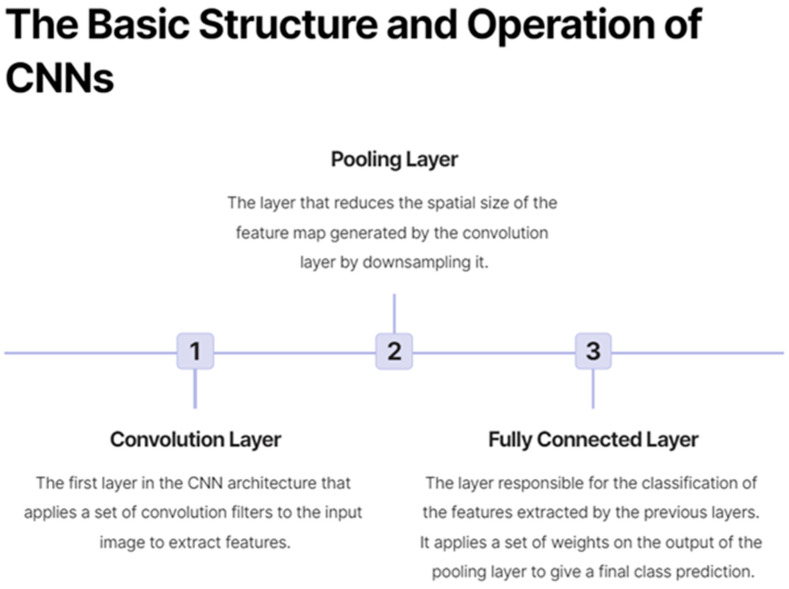
On the basic structure and operation of convolutional neural networks (CNNs).

Deep learning is a subfield of machine learning that involves the use of artificial neural networks with multiple layers, also known as deep neural networks, to automatically learn hierarchical representations of input data [15]. This advanced computational approach has shown remarkable success in various domains, including natural language processing, speech recognition, and computer vision. Convolutional neural networks (CNNs) are a type of deep learning architecture specifically designed for image analysis. They have demonstrated exceptional performance in various computer vision tasks, such as image classification, object detection, and segmentation. Figure 1 shows a lymph node metastasis segmented by a neural network algorithm. CNNs typically consist of multiple layers, including convolutional, pooling, and fully connected layers, that work together to automatically learn features and representations from raw image data, making them particularly suitable for medical image analysis, as shown in Figure 2. Some CNNs also include additional layers such as upsampling, which can be useful for image segmentation tasks.

### 3.2. Deep Learning in Medical Image Analysis: Performance Improvements over Traditional Methods

The application of deep learning techniques to medical image analysis has shown promising results in various domains, leading to significant advancements in the detection and diagnosis of diseases, the segmentation of anatomical structures, and the prediction of treatment outcomes [16]. Deep learning algorithms can learn complex patterns from medical images, generalizing well to new data and achieving human-level or even superior performance in many tasks [17]. This ability offers the potential to greatly improve the accuracy, efficiency, and consistency of medical image analysis, ultimately benefiting patient care and outcomes.

Quantitative comparisons between deep learning models and traditional pre-deep learning imaging techniques have demonstrated substantial improvements in performance. For instance, in the diagnosis of diabetic retinopathy from retinal images, deep learning models have achieved sensitivity of 96.8% and specificity of 87.0%, significantly surpassing traditional methods with sensitivities of 49.3–85.5% and specificities of 71.0–93.4% [18]. Similarly, deep learning-based detection of pulmonary nodules on CT scans has exhibited higher accuracy (94.2%) than conventional computer-aided detection methods (79.1%) [19]. In the segmentation of brain tumors from MRI scans, deep learning models have achieved a Dice similarity coefficient of 0.88, outperforming traditional methods with coefficients ranging from 0.65 to 0.85 [20].

**Figure 3:**
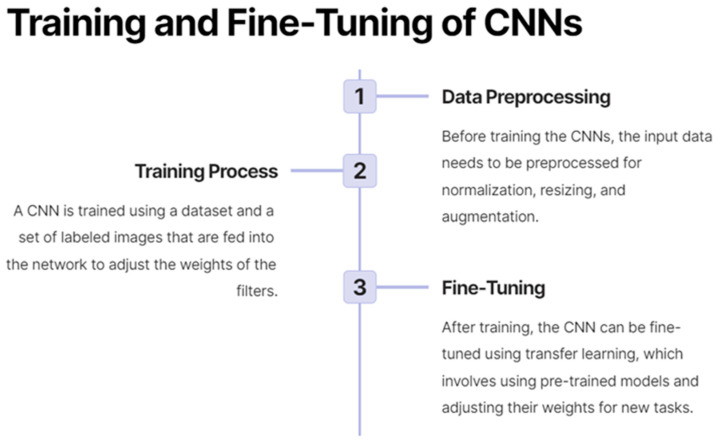
On the training and fine-tuning of convolutional neural networks (CNNs).

Moreover, deep learning has enabled the development of models that can integrate multi-modal and multi-scale imaging data, as well as clinical and demographic information, to generate more accurate and comprehensive predictions for patient outcomes and treatment responses [17]. This ability has contributed to the growing field of radiomics, which aims to extract and analyze high-dimensional quantitative features from medical images to build predictive models for personalized medicine [21]. As a result, deep learning is playing an increasingly important role in advancing precision medicine and improving patient care across a range of diseases and medical conditions. Figure 3 demonstrates the process by which CNNs are trained and fine-tuned.

## 4. Deep Learning in Head and Neck Cancer Imaging

### 4.1. MRI

Magnetic resonance imaging (MRI) is a non-invasive imaging modality that provides excellent soft tissue contrast and high spatial resolution, making it a valuable tool for the assessment of head and neck cancers [22]. Deep learning techniques have been applied to various tasks in head and neck MRI, ranging from tumor detection and segmentation to treatment response prediction and prognosis assessment.

#### 4.1.1. Tumor Detection and Segmentation

Convolutional neural networks (CNNs) have been employed for the automated detection and segmentation of head and neck tumors on MRI scans [23]. Recent studies have reported promising results in segmenting primary tumors and lymph nodes in patients with oropharyngeal cancer [24]. For instance, Men et al. (2017) reported a Dice similarity coefficient (DSC) of 0.85 for primary tumor segmentation using a 3D CNN, compared to a DSC of 0.74 for an atlas-based approach [25]. Advanced CNN architectures, such as U-Net and its variants, have been particularly successful in these segmentation tasks [26].

However, challenges remain in segmenting tumors with irregular shapes and heterogeneous intensity profiles, which may require the development of more sophisticated deep learning models or the incorporation of additional information, such as clinical or demographic data, to improve segmentation performance [5].

#### 4.1.2. Treatment Response Prediction and Prognosis Assessment

Deep learning models have also shown potential in predicting treatment response in head and neck cancer patients using MRI scans. Recent studies have demonstrated the ability of CNNs to predict outcomes, such as tumor regression, local control, and overall survival, from pre- and post-treatment MRI scans for various forms of cancer [27,28]. Some research in other forms of cancer has explored the use of multi-parametric MRI, combining multiple imaging sequences to improve the accuracy of treatment response prediction [29].

Radiomics, which involves the extraction of high-dimensional features from medical images, has been combined with deep learning for improved prediction of treatment response and prognosis [6]. This approach can lead to more personalized treatment plans and better monitoring of patient outcomes. However, the clinical utility of these models remains to be established through large-scale, prospective studies.

### 4.2. CT

Computed tomography (CT) scans are widely used in the diagnosis and staging of head and neck cancers due to their high spatial resolution, fast acquisition times, and ability to visualize bony structures [30]. Deep learning applications in head and neck cancer CT imaging include tumor detection and segmentation, outcome prediction, and treatment planning.

#### 4.2.1. Tumor Detection and Segmentation

Several studies have reported the use of CNNs for the detection and segmentation of head and neck tumors in CT scans [31,32]. CNNs have demonstrated high accuracy in segmenting primary tumors and lymph nodes, as well as in delineating organs at risk, such as the spinal cord and parotid glands, for radiotherapy planning [16,25,31].

Techniques such as 3D CNNs and multi-scale learning have been employed to better capture the spatial relationships between structures and to improve segmentation performance [33].

However, the performance of these models can be affected by the presence of artifacts, such as metal implants and dental fillings, which are common on head and neck CT scans [34]. Future research may explore the use of deep learning techniques for artifact reduction or may develop models that are more robust to artifacts in the image data.

#### 4.2.2. Outcome Prediction and Treatment Planning

Deep learning models have also been developed for predicting treatment outcomes in head and neck cancer patients using CT scans. A recent study demonstrated the ability of a CNN to predict overall survival and disease-free survival from pre-treatment CT scans in patients with nasopharyngeal carcinoma [35]. Moreover, deep learning models incorporating radiomic features have shown improved prediction of treatment response and survival [33].

In addition, deep learning has been applied to treatment planning, including the optimization of radiotherapy plans, for head and neck cancer patients. These models can automatically generate dose distribution predictions and identify optimal beam configurations to minimize radiation exposure to healthy tissues while maximizing tumor coverage [36]. However, the generalizability and clinical impact of these models warrant further investigation, including multi-center studies and the evaluation of their performance in diverse patient populations.

### 4.3. PET

Positron emission tomography (PET) scans, which provide functional information about tumor metabolism and tissue perfusion, have gained increasing importance in the management of head and neck cancers [37]. Deep learning techniques have been applied to PET scans for tasks such as tumor detection, segmentation, and outcome prediction.

#### 4.3.1. Tumor Detection and Segmentation

CNNs have shown promising results in the detection and segmentation of head and neck tumors on PET scans [24]. Methods such as transfer learning and the use of multi-modal data, combining PET with CT, or MRI, have been explored to improve the performance of deep learning models in this domain [38].

However, the performance of these models can be affected by the low spatial resolution and high noise levels inherent to PET imaging, as well as variations in image acquisition protocols and reconstruction algorithms [39]. Future research should focus on developing models that are more robust to these challenges and investigating strategies for harmonizing PET data from different sources.

#### 4.3.2. Outcome Prediction and Treatment Monitoring

Deep learning models have also been explored for predicting treatment outcomes in head and neck cancer patients using PET scans. Studies have reported the potential of CNNs to predict tumor response, recurrence, and survival from pre- and post-treatment PET scans [40]. Some research has also examined the use of PET-derived radiomic features in combination with deep learning models for improved outcome prediction [41].

Furthermore, deep learning has been applied to monitor treatment response in head and neck cancer patients using PET scans. These models can potentially identify early metabolic changes that indicate treatment effectiveness, allowing for timely adjustments to treatment plans and improved patient outcomes [42]. However, further research is needed to validate these models in large, prospective cohorts and to establish their clinical utility.

## 5. Comparison of Deep Learning and Traditional Imaging Techniques in Head and Neck Cancer Imaging

While deep learning techniques have shown promise in various head and neck cancer imaging tasks, it is crucial to consider the specific situations in which traditional imaging techniques remain valuable and those in which deep learning tools are more appropriate.

### 5.1. Advantages of Traditional Imaging Techniques

1. Interpretability: Traditional imaging techniques provide interpretable results, as they are often based on well-established, handcrafted features and statistical methods [43]. This interpretability allows clinicians to better understand the rationale behind the decision-making process, which is essential for building trust in the results and ensuring appropriate clinical actions.
2. Lower computational requirements: Traditional imaging methods typically have lower computational demands compared to deep learning approaches [16], making them more accessible and easier to implement on standard workstations without the need for high-performance computing resources.
3. Robustness to variations: Traditional imaging techniques may be more robust to variations in imaging protocols and acquisition parameters [44] since they rely on well-established features that are less sensitive to changes in image quality and appearance.

### 5.2. Advantages of Deep Learning Techniques

1. Improved accuracy: Deep learning techniques, particularly CNNs, have demonstrated superior performance in many head and neck cancer imaging tasks, including tumor detection, segmentation, and outcome prediction, compared to traditional methods [25,27,45].
2. Automatic feature learning: Deep learning models can automatically learn relevant features from the data without the need for manual feature engineering [5], reducing the potential for human bias and enabling the discovery of novel imaging biomarkers that may not be apparent using traditional techniques.
3. Integration of multi-modal and multi-parametric data: Deep learning models can efficiently manage and integrate information from various imaging modalities (e.g., MRI, CT, and PET) and different image sequences [16], potentially providing a more comprehensive assessment of tumor characteristics and treatment response.

### 5.3. Guidance for Optimal Use

1. Preliminary analysis and simpler tasks: In situations in which a quick, preliminary analysis is required or for less complex tasks, traditional imaging techniques may be more suitable due to their lower computational demands and ease of implementation [16].
2. Interpretability and trust: When interpretability and trust in the decision-making process are critical, traditional methods may be more appropriate, as they provide more transparent and explainable results [46].
3. Resource-limited settings: In settings in which computational resources are limited, traditional imaging techniques may be more feasible since they generally have lower computational requirements [16].
4. Complex tasks and improved performance: For more complex tasks or when seeking improved accuracy and performance, deep learning techniques are more suitable [24,25,27,45]. This suitability includes for tasks such as tumor detection and segmentation, outcome prediction, and treatment planning in head and neck cancer imaging.

By carefully considering the specific situation and the desired outcomes, clinicians and researchers can select the most appropriate method for achieving accurate and reliable results in head and neck cancer imaging.

## 6. Gaps, Controversies, and Future Directions

### 6.1. Lack of Standardization and Benchmarking

One major challenge in the field of deep learning for head and neck cancer imaging is the lack of standardization and benchmarking. Diverse datasets, preprocessing techniques, model architectures, and training strategies have been used in different studies, making it difficult to compare the performance of various models and to assess their clinical utility [38]. Inconsistent reporting of model performance metrics, such as accuracy, sensitivity, and specificity, further complicates the comparison of results across studies. Future research should focus on establishing standardized datasets, evaluation metrics, and reporting guidelines to facilitate the benchmarking and comparison of deep learning models. In addition, promoting open science practices, such as sharing of data and code, can help to accelerate the development and validation of deep learning models for head and neck cancer imaging. 

### 6.2. Integration of Multimodal, Temporal, and Clinical Information

Most deep learning studies in head and neck cancer imaging have focused on single-modality imaging data, such as MRI, CT, or PET scans. However, integrating multimodal information, such as combining functional and anatomical imaging data, can potentially improve the performance of deep learning models and provide a more comprehensive understanding of tumor characteristics [39]. Moreover, incorporating temporal information from longitudinal imaging data can potentially enhance the prediction of treatment response, tumor recurrence, and patient outcomes [40]. In addition to imaging data, the integration of clinical information, such as patient demographics, tumor histology, and treatment details, can further improve the performance of deep learning models in head and neck cancer management. Future research should explore the development of deep learning models that can effectively integrate multimodal, temporal, and clinical information for personalized treatment planning and prognostication. 

### 6.3. Explainability, Interpretability, and Trustworthiness

Deep learning models, particularly CNNs, are often considered “black boxes” due to their complex architectures and the lack of transparency in the decision-making process [47]. This lack of transparency can hinder the adoption of deep learning techniques in clinical practice since clinicians may be reluctant to trust a model’s predictions without understanding the underlying reasoning. Developing explainable and interpretable deep learning models is crucial to bridge this gap and promote their acceptance in the medical community [41]. Techniques such as attention mechanisms, layer-wise relevance propagation, and visualization of feature maps can help to elucidate the reasoning behind a model’s predictions and build trust among clinicians. Future research should focus on incorporating explainability and interpretability into the design of deep learning models for head and neck cancer imaging, as well as on developing methods to assess the trustworthiness and robustness of these models in the face of noisy, incomplete, or adversarial data.

### 6.4. Clinical Implementation, Validation, and Impact Assessment

The translation of deep learning models from research to clinical practice requires rigorous validation and assessment of their impact on patient outcomes [42]. Large-scale, prospective studies would be useful to establish the performance, generalizability, and clinical utility of deep learning models for head and neck cancer imaging [25]. These studies should involve diverse patient populations and imaging data from multiple centers to ensure the robustness of the models in real-world settings. Moreover, the integration of these models into existing clinical workflows, the assessment of their cost-effectiveness, and the evaluation of their impact on patient care, such as the reduction in diagnostic errors, optimization of treatment planning, and improvement of patient outcomes, are essential steps toward their successful implementation [48]. Future research should also explore the development of user-friendly, scalable, and secure software tools and platforms that can facilitate the deployment of deep learning models in clinical settings and enable their widespread adoption in head and neck cancer management.

## 7. Challenges and Limitations of Deep Learning Models in Head and Neck Cancer Imaging

### 7.1. Data Quality and Quantity

The performance of deep learning models heavily relies on the quality and quantity of the training data [5]. Obtaining large, diverse, and well-annotated datasets for head and neck cancer imaging can be challenging due to factors such as privacy concerns, data sharing restrictions, and the time-consuming nature of manual annotation by experts [49]. Additionally, variations in imaging protocols, scanner types, and image acquisition parameters across different institutions can lead to inconsistencies in the data, affecting model performance [50]. Future research should focus on developing methods to leverage smaller or imperfect datasets, explore data augmentation techniques, and promote multi-institutional collaboration to enhance the performance of deep learning models. 

### 7.2. Model Overfitting

Deep learning models are prone to overfitting, especially when trained on small datasets, leading to poor generalization to new data [6]. Overfitting can result in models that perform well on the training data but fail to accurately predict outcomes for new patients, limiting their clinical utility. Regularization techniques and model architectures that mitigate overfitting, such as dropout, batch normalization, and transfer learning, should be considered when developing deep learning models for head and neck cancer imaging [51]. Moreover, the use of cross-validation and external validation cohorts can help to evaluate and improve model generalizability.

### 7.3. Computation Requirements

The training and deployment of deep learning models often require substantial computational resources, such as graphics processing units (GPUs) and specialized hardware [5]. This need may pose a challenge for small clinical centers and researchers with limited access to high-performance computing facilities. Developing efficient model architectures, exploring strategies for model compression and acceleration, and utilizing cloud-based platforms for training and deployment can help to overcome these challenges and make deep learning models more accessible to a broader range of institutions and researchers.

### 7.4. Ethical Considerations

The use of deep learning models in medical imaging raises several ethical concerns, including those concerning data privacy, algorithmic fairness, and accountability [52]. Ensuring the protection of patient data is paramount, and researchers should adhere to data protection regulations and employ techniques such as data anonymization and secure data storage to safeguard patient privacy [53]. Moreover, biases in training data can lead to unfair treatment recommendations or misdiagnoses for certain patient populations, making it essential to address potential biases in the development and deployment of deep learning models. Establishing transparent and accountable practices, such as reporting model performance across diverse patient groups and involving stakeholders in the model development process can help to ensure ethical and responsible use of these technologies in head and neck cancer imaging.

## 8. Radiogenomics: A Promising Avenue for Deep Learning in Head and Neck Cancer Imaging

### 8.1. Radiogenomics: An Overview

Radiogenomics is an emerging interdisciplinary field that investigates the relationship between imaging features and genomic information, with the goal of developing predictive models that can guide personalized treatment decisions [10]. By integrating radiogenomic data with deep learning models, researchers and clinicians can gain a more comprehensive understanding of tumor biology, heterogeneity, and treatment response, ultimately leading to improved patient outcomes in head and neck cancer management. 

### 8.2. Radiogenomic Features in Deep Learning Models

Several studies have explored the incorporation of radiogenomic features into deep learning models for head and neck cancer imaging [25,33]. Combining radiomic features extracted from MRI, CT, or PET scans with genomic data, such as gene expression profiles or mutational status, can improve the accuracy of tumor detection, segmentation, and outcome prediction [54]. Additionally, deep learning models can be designed to automatically learn radiogenomic representations from the imaging data, potentially revealing novel imaging-genomic associations that may have clinical implications [54]. This approach could lead to the identification of previously unknown biomarkers and therapeutic targets in head and neck cancers.

### 8.3. Challenges and Future Directions

Despite the promising potential of radiogenomics in deep learning for head and neck cancer imaging, several challenges need to be addressed. First, the acquisition of genomic data can be costly and invasive, limiting their availability in clinical settings [55]. Non-invasive methods, such as liquid biopsy, may offer a more accessible source of genomic information in the future [56]. Second, the integration of imaging and genomic data presents computational challenges since these data types have different scales and dimensions [10]. Developing novel deep learning architectures and data fusion techniques that can effectively combine and process multimodal data is a crucial area for future research. Additionally, addressing the issues of data heterogeneity, missing data, and standardization of imaging and genomic data will be essential for the successful application of radiogenomics in deep learning models [57].

### 8.4. Clinical Implementation and Validation

As with any novel approach in medical imaging, the translation of radiogenomic-based deep learning models to clinical practice requires rigorous validation and assessment of their impact on patient outcomes. Large-scale, multi-institutional studies would be useful to establish the performance, generalizability, and clinical utility of these models in head and neck cancer management [16]. Furthermore, the integration of radiogenomic-based models into existing clinical workflows and the assessment of their cost-effectiveness are essential steps toward their successful implementation. As radiogenomics continues to evolve, it is expected that deep learning models incorporating radiogenomic features will play an increasingly important role in guiding personalized treatment strategies for head and neck cancer patients.

## 9. Advanced Deep Learning Techniques in Head and Neck Cancer Imaging

### 9.1. Convolutional Autoencoders for Data Compression, Image Denoising, and Feature Extraction

Convolutional autoencoders (CAEs) are a type of deep learning model that can be used for unsupervised feature learning, image denoising, and data compression [58]. They consist of an encoder network, which reduces the input image to a compact representation, and a decoder network, which reconstructs the image from this representation. CAEs have been applied in various medical imaging tasks, including head and neck cancer imaging, for improving image quality and reducing noise, as well as extracting relevant features that can be used in downstream analysis [59,60].

### 9.2. Generative Adversarial Networks for Super-Resolution

Generative adversarial networks (GANs) are a class of deep learning models that consist of two competing networks: a generator and a discriminator [61]. The generator creates synthetic images, while the discriminator evaluates the quality of these images, aiming to distinguish between real and generated images. GANs have been used for various medical image enhancement tasks, such as super-resolution, in which the goal is to generate high-resolution images from low-resolution input data. In the context of head and neck cancer imaging, GANs can be utilized to improve image resolution, potentially leading to more accurate tumor detection and segmentation [62,63].

### 9.3. Transformer Models: Vision Transformer (ViT)

Transformer models, initially introduced for natural language processing tasks, have been adapted for image analysis with the development of the Vision Transformer (ViT) [64]. ViT divides an image into non-overlapping patches, linearly embeds them into a sequence of fixed-size vectors, and processes these vectors using the transformer architecture. This approach allows the model to capture long-range dependencies and spatial relationships between image regions. ViT has shown competitive performance in various medical imaging tasks, including head and neck cancer detection and segmentation, and it can be considered an alternative to traditional CNN-based methods [65,66].

## 10. Conclusions

Deep learning techniques have made significant strides in the field of head and neck cancer imaging, demonstrating exceptional performance in critical tasks, such as tumor detection, segmentation, and outcome prediction, using various imaging modalities, such as MRI, CT, and PET scans. Advanced deep learning techniques, including convolutional autoencoders, generative adversarial networks (GANs), and transformer models such as the Vision Transformer (ViT), have further enhanced the capabilities of these models, offering promising improvements in data compression, image denoising, super-resolution, and feature extraction. The incorporation of radiogenomics into deep learning models offers a promising avenue for further advancements, potentially enabling a more comprehensive understanding of tumor biology and heterogeneity and guiding personalized treatment strategies in head and neck cancer management.

Despite these encouraging results, a number of challenges must be addressed to facilitate the successful implementation of deep learning models in head and neck cancer diagnosis and treatment. One major obstacle is the lack of standardization in terms of datasets, preprocessing techniques, and model architectures, rendering it difficult to compare the performances of various models and assess their clinical utility. Additionally, acquiring large, diverse, and well-annotated datasets for head and neck cancer imaging poses a challenge due to privacy concerns, data sharing restrictions, and the labor-intensive nature of manual annotation by experts.

In light of the limitations of traditional imaging techniques, such as subjectivity and variability in human interpretation, the integration of deep learning and traditional imaging techniques could lead to more robust and accurate diagnosis and treatment planning. Recognizing the complementary roles of these approaches is essential for advancing head and neck cancer imaging and management.

Model overfitting, a common issue in deep learning, is another concern, particularly when models are trained on small datasets. This situation can lead to poor generalization from new data and reduced clinical utility. Moreover, the computational requirements for training and deploying deep learning models can be substantial, often necessitating specialized hardware and resources that may not be readily accessible to smaller clinical centers or researchers. Addressing these challenges, along with integrating multimodal and temporal information from different imaging modalities and timepoints, is crucial for further enhancing the performance of deep learning models. Developing explainable and interpretable models is also essential to bridge the gap between complex model architectures and clinical decision-making, fostering trust and acceptance among medical professionals.

Finally, conducting rigorous clinical validations through large-scale, prospective studies is necessary to establish the performance, generalizability, and clinical utility of deep learning models for head and neck cancer imaging. Overcoming these obstacles, while embracing the synergy between advanced deep learning techniques and traditional imaging approaches, will be instrumental in paving the way for improved patient outcomes and the realization of personalized treatment strategies in head and neck cancer management.
